# Tongue cancer with mental retardation due to microcephaly: a clinical report

**DOI:** 10.1186/s12957-015-0634-2

**Published:** 2015-07-16

**Authors:** Akifumi Enomoto, Takanori Nakatani, Eri Morikage, Takeshi Shimoide, Suguru Hamada

**Affiliations:** Department of Oral and Maxillofacial Surgery, Kinki University School of Medicine, 377-2, Onohigashi, Osaka-Sayama, 589-8511 Japan

**Keywords:** Tongue cancer, Mental retardation

## Abstract

Oral cancer in patients with mental retardation has not been reported in detail, although the literature on clinical management of oral malignancies in the general population is extensive. No clear consensus has been established regarding the management of oral cancer in patients with mental retardation. We present herein the case of a 32-year-old Japanese man with mental retardation due to microcephaly who presented with advanced tongue cancer. He was treated with three courses of chemotherapy using superselective intra-arterial infusion of cisplatin at 100 mg/m^2^ via the femoral artery (Seldinger method). No major complications were encountered, and complete response was achieved. The patient has shown no clinical or radiological evidence of local recurrence or distant metastases as of 22 months after the end of treatment. This case provides a basis for the future appropriate management of oral cancer in patients with mental retardation.

## Background

The literature on the clinical management of oral malignancies in the general population is extensive. Diagnosis and management planning for the staging of patients with squamous cell carcinoma (SCC) of the head and neck region has increasingly been reported. However, issues associated with oral cancer in patients with mental retardation have not been widely discussed, since the importance of environmental and constitutional risk factors associated with mental retardation are unclear. Due to the difficulties in communicating about their own oral health, achieving the cooperation of the patient with the treatment procedure is always complicated. In addition, poor ability in communication can delay the diagnosis of oral cancer. To improve the lack of information on oral cancer in patients with mental retardation, further reports are required. We present a case of tongue cancer in a patient with mental retardation due to microcephaly. This research was approved by the review board of Kinki University School of Medicine.

## Case presentation

**Fig. 1:**
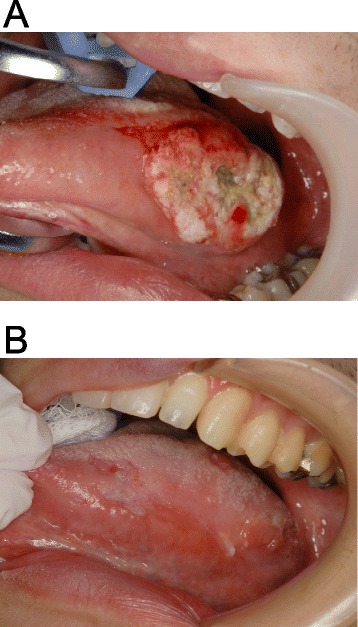
Intraoral findings. Intraoral photographs at initial presentation (**a**) and at 22 months after treatment (**b**). Advanced cancer is seen in the left lateral tongue with marked swelling

**Fig. 2:**
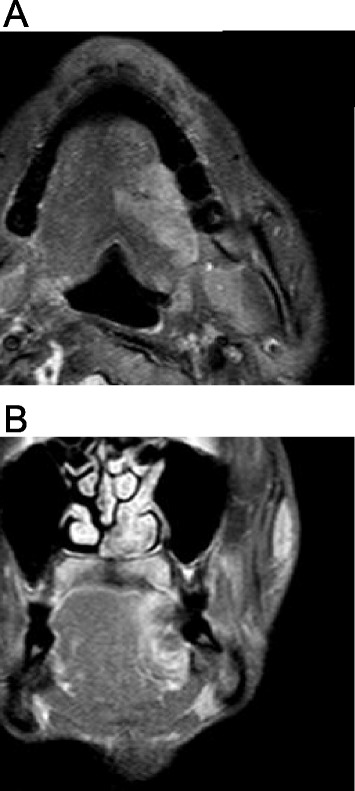
MRI assessment at initial examination. STIR (**a**) and contrast-enhanced T1-weighted imaging (**b**) on initial presentation show a prominent hyperintense lesion in the left tongue

A 32-year-old Japanese man was referred with his parents to our clinic in August 2012 complaining of swelling of the left tongue. Considerable swelling in the left tongue was evident (Fig. 1a), but no lymph nodes were palpable on the either side of the neck. The patient had mental retardation due to microcephaly, and needed supportive care for all activities of daily living. Magnetic resonance imaging (MRI) and contrast-enhanced computed tomography (CT) were performed with intravenous sedation. On MRI, hyperintensity on short T1 inversion recovery (STIR) images and contrast-enhanced T1-weighted images (maximum length 42 mm) was observed in the left tongue (Fig. 2a, b). CT evaluation of the tongue was unclear due to a metal artifact caused by a dental prosthesis. Biopsy of the left tongue lesion was performed under intravenous sedation, revealing well-differentiated SCC. Finally, a diagnosis of SCC of the left tongue with no lymph node metastasis was established. The tumor was staged as T3N0M0 (stage III) according to the Union for International Cancer Control staging system.

**Fig. 3:**
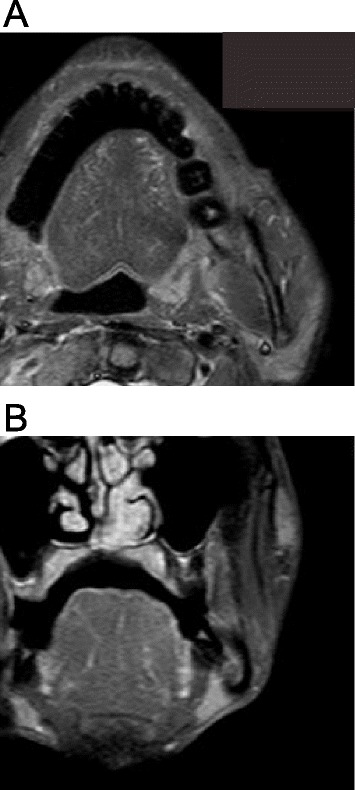
MRI assessment after treatment. STIR (**a**) and contrast-enhanced T1-weighted imaging (**b**) at 22 months after treatment show no apparent abnormal lesion in the corresponding area

After sufficient consultation with an interdisciplinary team and provision of informed consent by his family, chemotherapy was selected as the treatment strategy. Three courses of chemotherapy were administered using superselective intra-arterial infusion via the femoral artery (Seldinger method) with cisplatin (CDDP) at 100 mg/m^2^. The interval for chemotherapy was set at 2 weeks. During chemotherapy, the patient developed grade 1 stomatitis, grade 1 vomiting, and grade 2 neutropenia (National Cancer Institute common toxicity criteria, version 3.0). MRI assessment obtained 2 weeks after completing the treatment course demonstrated dramatic regression of the tumor. After 22 months, the treatment was deemed to have achieved complete response (CR) (Figs. 1b and 3a, b).

This represents the first description of advanced tongue cancer in a patient with mental retardation treated with chemotherapy using superselective intra-arterial infusion by the Seldinger method.

The incidence of cancer in individuals with mental retardation is reportedly as frequent as in the general population, although the frequency of oral cancer with mental retardation may be slightly reduced because of reduced exposures to risk factors for oral SCC such as tobacco and alcohol [1–5]. However, treatment strategies for oral cancer in patients with mental retardation are unclear, with only a few studies reported [1, 6]. As far as we have been able to determine from a PubMed search of the literature from the past 30 years, 11 cases of tongue cancer among individuals with mental retardation have been reported, including the present case [6–12]. The prognosis for these cancer patients seems unfavorable (Table 1). In addition, information on the treatment process is lacking, with descriptions in only a small number of reports. Further information is thus required.

**Table 1:** Review of the literature: 11 cases of tongue cancers in patients with mental retardation

Authors’ prognosis	Sex/age	Associated disease	TNM	Treatment	Follow-up term
Jancar et al. [10] NA	F/60	Cockayne syndrome	NA	NA	NA
Farhat et al. [8] DOD	M/27	Down syndrome	T2N0M0	S/CRT	1 year
Sund et al. [6] NA	F/54	Fragile X syndrome	NA	NA	NA
Kiani et al. [11] DOD	F/NA	NA	NA	NA	NA
Hennequin et al. [9]	NA	Down syndrome	NA	NA	NA
DOD					
DOD	NA	Down syndrome	NA	NA	NA
Satge et al. [5] DOD	F/NA	NA	NA	NA	NA
Butt et al. [7] NA	M/17	XP	NA	NA	NA
Alive at 21 years	M/20	XP	NA	S	1 year
NA	M/11	XP	T1NxM1	S	6 months
Enomoto et al. Alive at 34 years	M/32	Microcephaly	T3N0M0	CT	2 years

*TNM* TNM classification, *NA* not available, *DOD* dead of disease, *XP* xeroderma pigmentosa, *S* surgery, *CRT* chemoradiotherapy, *CT* chemotherapy

One of the major factors is that treatment for oral cancer in patients with mental retardation is difficult due to the impaired physiological situation based on the biological background, as well as difficulties with understanding and communication. In addition, the poor ability of these patients in communicating about their own health situation may delay the diagnosis of oral cancer, as evidenced by the delayed discovery and diagnosis of advanced tongue cancer in the present case. Generally, a high survival rate cannot be expected with advanced oral cancer (stage III or IV), unlike stage I or II oral cancer. Surgery and postoperative radiotherapy and/or chemotherapy are thus suggested as standard treatments. These treatments, however, have major side effects such as impaired swallowing and pronunciation problems due to the resulting physiological changes, or the development of oral stomatitis. Considering the standard treatment of oral cancer, particularly for tongue cancer, impaired swallowing ability or postoperative tracheal anomalies resulting from surgery are unacceptable for patients with mental retardation [1]. Surgery or anesthesia will occasionally be inadequate because of biological conditions such as atlantoaxial instability or cardiomyopathy. Daily treatments requiring the body to be kept still for long periods in radiotherapy may be difficult, and predictable development of oral stomatitis can be intolerable for patients with tongue cancer. Chemotherapy also needs modification of the protocol, in accordance with the biochemical and metabolic condition of the patient. In these regards, chemotherapy could be adapted to patients with mental retardation, as previously reported [1]. The efficacy and advantages of superselective intra-arterial chemotherapy for head and neck cancer have recently been reported in several papers [13–17], and infusion using the Seldinger method proved adequate in the present case. Chemotherapy with CDDP on day 1 using the Seldinger method was thought to be suitable for this patient, while daily concurrent chemotherapy with infusion via a superficial temporal artery may have been unacceptable and difficult. In this patient, three courses of chemotherapy were administered, each under general anesthesia. Since general anesthesia is often needed for dental treatments in patients with mental retardation due to their difficulty in communicating with medical staff, general anesthesia appeared reasonable in the present case.

After 22 months, the treatment was deemed to have achieved CR, although further observation is required. Chemotherapy using superselective intra-arterial infusion by the Seldinger method with CDDP was therefore effective as treatment for the present patient.

The treatment strategy for oral cancer in patients with mental retardation is difficult and poorly known in many aspects. A multidisciplinary approach should thus be required, and further experiences need to be accumulated and shared.

## Conclusions

We present the case of mental retardation patient with advanced tongue cancer. The three courses of chemotherapy using superselective intra-arterial infusion of cisplatin via the femoral artery (Seldinger method) were performed. The patient has shown no clinical or radiological evidence of local recurrence or distant metastases as of 22 months after the end of treatment.

## Consent

Written informed consent was obtained from the patient for publication of this case report and any accompanying images.
